# An Evolutionary Insight Into the Heterogeneous Severity Pattern of the SARS-CoV-2 Infection

**DOI:** 10.3389/fgene.2022.859508

**Published:** 2022-03-22

**Authors:** Rabail Zehra Raza, Sumra Wajid Abbasi

**Affiliations:** NUMS Department of Biological Sciences, Faculty of Multidisciplinary Studies, National University of Medical Sciences, Rawalpindi, Pakistan

**Keywords:** COVID-19, host genetic factors, natural selection, population genomics, SNP variants

## Abstract

The ongoing pandemic of COVID-19 has elaborated an idiosyncratic pattern of SARS-CoV-2-induced symptoms in the human host. Some populations have succumbed to the SARS-CoV-2 infection in large numbers during this pandemic, whereas others have shown a resilient side by manifesting only milder or no symptoms at all. This observation has relayed the onus of the heterogeneous pattern of SARS-CoV-2-induced critical illness among different populations to the host genetic factors. Here, the evolutionary route was explored and three genetic loci, i.e., rs10735079, rs2109069, and rs2236757, associated with COVID-19 were analyzed. Among the three, the risk allele A at genetic locus rs2236757 residing in the *IFNAR2* gene was observed to have undergone recent positive selection in the African population.

## Introduction

Coronaviruses have been around for the last 2 decades and were declared pathogenic to humans in the early 21st century after the first severe acute respiratory syndrome (SARS) outbreak (Cui et al., 2019). The recent worldwide surge in the novel SARS-CoV-2 infection during 2020 has made it a global pandemic. SARS-CoV-2 has a single-stranded RNA in its genome which depends on RNA-dependent RNA polymerase for its replication (Siqueira et al., 2021). RNA viruses are prone to mutations. The more the RNA virus replicates, the more changes it accumulates in the genome because of a lack of proofreading polymerase activity (Shen et al., 2020). Because of this rapid intra-host replication, highly related viral entities of RNA viruses (quasi species) arise in the infected host (Siqueira et al., 2021). Within-host evolution of viruses has previously been reported for many RNA viruses such as MERS, SARS-CoV-1 and influenza (Xue et al., 2018; Al Khatib et al., 2020). In the case of COVID-19, Shen et al. (2020) identified 0 to 51 viral entities per hospitalized COVID-19 patient from the Chinese District, Wuhan, in December 2019. The SARS-CoV-2 quasi species has also been analyzed in relation to disease severity in COVID-19 patients. One such study reported significant diversity in SARS-CoV-2 genomes at the sub-consensus sequence level between mild and severe patients and observed a considerable increase in the number of coding and non-coding variants in severe cases as compared to the mild ones (Al Khatib et al., 2020). However, scarcity of significant variation in SARS-CoV-2 genomes at the consensus level (where similarity of all viral sequences is greater than 99.8%) has led scientists to believe that the host genetic factors, for instance, age, gender, and other underlying comorbidities, along with environmental and social factors, play a vital role in determining COVID-19 severity among patients (Guan et al., 2020).

The World Health Organization (WHO) has reported more than 100 million confirmed cases of COVID-19 across 223 countries since the start of the pandemic. The phenotypic results of the SARS-CoV-2 infection are in stark contrast, with some patients showing mild to no visible symptoms and others undergoing fatal respiratory distress (Siqueira et al., 2021). In multiple studies, people with male gender, older age, smoking history, cancer, and other underlying comorbidities such as obesity, hypertension, and autoimmune disorders have been identified as vulnerable groups to getting severely infected with SARS-CoV-2 (Atkins et al., 2020). Although a broader risk group for COVID-19 mortality with pre-existing comorbidities has been identified, the dilemma of idiosyncratic symptomatic responses to SARS-CoV-2 infection in otherwise healthy patients is still under discussion (Hu et al., 2020; Williamson et al., 2020). It also remains a conundrum as to why certain populations have shown a much greater mortality rate associated with COVID-19 than others. For instance, in Africa, the number of deaths reported from SARS-CoV-2 infection was predicted to be much higher given the continent’s higher population density, weaker healthcare systems, lower finances, and lack of preparedness in the wake of a global pandemic (Mbow et al., 2020; Maeda and Nkengasong, 2021). However, on the contrary, the number of COVID-19 deaths reported in Africa has been much lower than expected. According to the Africa CDC, the number of COVD-19 deaths till November 2020, made up 3.6% of the total worldwide cases (https://africacdc.org/covid-19) (Maeda and Nkengasong, 2021). In the recent upsurge of the OMICRON crisis in Africa, the casualty rate has surpassed 0.2 million by early 2022, as reported by the Africa CDC (https://africacdc.org/covid-19), which is not equal to even half of the casualties (0.86 million) reported from the US alone because of the SARS-CoV-2 pandemic. Although myriad reasons could be called upon for populations who seemingly did not get affected by COVID-19 as much as others, such as poor reporting, testing, and having a younger population, to name a few, the fickle nature of the symptoms among the same human host at different geographical distributions needs a robust investigation (Chitungo et al., 2020). Various aspects of the COVID-19 host-specific severity have been explored, of which rapid mutations in the SARS-CoV-2 RNA genome have also been taken into account between the severe and milder cases. However, the results do not suffice the answer as to why some populations showed a greater casualty rate.

To gauge the disparity in the number of COVID-19 deaths among different populations or even between the individuals of the same population, several studies have put forth the significance of within-host diversity of SARS-CoV-2 genomes between mild and severe cases of COVID-19 (Al Khatib et al., 2020; Shen et al., 2020). Within-host diversity of SARS-CoV-2 genomes has been determined at the consensus and sub-consensus levels in mild and severe cases of COVID-19. Although the within-host diversity of SARS-CoV-2 genomes has been identified at the sub-consensus level, indicating more variants in the SARS-CoV-2 genomes in severe cases, the importance of host genetic factors in creating erratic immune responses to the SARS-CoV-2 infection in some individuals cannot be ignored. Therefore, host genetic factors are deemed crucially important in the case of the COVID-19 severity conundrum among the populations. In order to analyze the heterogeneous trend of COVID-19 severity, evolution of the host genome with regard to COVID-19-associated genetic loci in different populations could show promising results. In this study, population-wise haplotype-based analysis was conducted by employing 1000 Genomes phase III data on three genetic loci associated with COVID-19 and signatures of selection on them were analyzed (Nature, 2015).

## Materials and Methods

### Data Collection

In this study, two GWAS studies conducted for COVID-19 associations meeting the genome-wide significance threshold (P-value < 5 × 10^−8^) were referred to (Group, 2020; Pairo-Castineira et al., 2020). Among the two studies, the older investigation published in June 2020 identified the association of two SNPs, rs11385942 (INDEL: INsertion-DELetion) and rs657152 (SNV: single nucleotide variant) with COVID-19 in a European cohort (Italian and Spanish). The former SNP rs11385942 with a genome-wide association P-value = 1.15 × 10^−10^ was located in a chromosomal location harboring many immunity-related genes such as *CXCR6*, *CCR1* and *CCR2* in close proximity (Group, 2020). The latter SNP rs657152 (A > C) is situated in the ABO blood group locus with a P-value = 4.95 × 10^−8^ in the meta-analysis (Group, 2020). The second GWAS study was published in December 2020 after investigating the critical care patients of the UK and identified associations of three SNPs, rs10735079 (SNV: A > G, P-value = 1.65 × 10^−8^), rs2109069 (SNV: A > G, P-value = 3.98 × 10^−12^), and rs2236757 (SNV: G > A, P-value = 4.99 × 10^−8^) with critical COVID-19-induced illness (Pairo-Castineira et al., 2020). Among the three SNPs, the neighboring genes such as *IFNAR2* and *OAS* genes are the immunity-related genes involved in the innate anti-viral defense response by the host (Pairo-Castineira et al., 2020).

### 1000 Genomes Phase III SNP Data

In this study, the 1000 Genomes Phase III SNP data for the analysis was referred to. There were shortlisted three single nucleotide variations (SNVs) among the aforementioned COVID-19-associated SNPs with neighboring/flanking genes because of their immunity-related function, i.e., rs10735079 (A > G), rs2109069 (A > G) and rs2236757 (G > A) residing in the *OAS* gene cluster, within *DPP9* and within *IFNAR2*, respectively (Pairo-Castineira et al., 2020). Because of the limitation that only SNVs can be used for haplotype-based tests in this study, it was not shortlisted for analysis even though the genes lying within the vicinity have an immunity-related function (Group, 2020). In order to collect the SNP data for a regional analysis of length as long as 1 Mb, VCF files pertaining to a 0.5 Mb region were collected on either side of the three aforementioned SNPs from the 1000 Genomes Phase III SNP data (Nature, 2015; Zehra et al., 2018). All three SNPs had a minor allele frequency ≥0.05 and were used to assess signals of positive selection by the subsequent haplotype-based tests in 2504 individuals of the 1000 Genomes Phase III data belonging to African, European, Asian, and American samples.

### Haplotype Based Selection Tests

To build a selection regime in a population, the two haplotypes of an individual, acquired from each parental chromosome, are necessary. This explains haplotype inference or phasing, a critical stage in population genetics research to separate the genotype information inherited from both parents (Salem et al., 2005). As phased haplotypes are needed to calculate the Extended Haplotype Homozygosity (EHH) test and haplotype bifurcation diagrams, the VCF files were first phased using fastPHASE to reconstruct haplotypes (Sabeti et al., 2002; Scheet and Stephens, 2006). EHH plots and haplotype bifurcation diagrams were made using the rehh package in R (Gautier and Vitalis, 2012). Furthermore, in order to gauge the genetic differentiation between the aforementioned subpopulations, Weir and Cockerham fixation index (F_st_) values were also evaluated using the VCFtools (Danecek et al., 2011). The F_st_ values ≥0.1 were considered significant. Moreover, Haploreg (version 4.1) and linkage disequilibrium (LD) calculator at the Ensembl genome browser were also used for corroborating the haplotype blocks of adjacent SNPs with LD (*r* ^2^) ≥ 0.8 that confirmed the long, unbroken haplotypes resulted by applying EHH test and the haplotype bifurcation diagrams (Ward and Kellis, 2012; Cunningham et al., 2015).

## Results and Discussion

Polymorphisms in the host genes such as *ACE2, TMPRSS2*, and *ADAM17* have been associated with their expression levels and ultimately influence the mechanism of SARS-CoV-2 infectivity and severity (Brest et al., 2020). In the human genome, mutations or genetic variants (alleles) on a locus can contribute to fitness and, because of the advantageousness they impart on the phenotypic fitness of the species, can undergo positive selection. Positive selection on beneficial alleles increases their frequency in a population, whereas negative selection discards the deleterious alleles (Karlsson et al., 2014). In a phenomenon known as linkage disequilibrium (LD), the signals of positive selection on a genomic position increase the frequency of the beneficial allele along with the neighboring alleles in a non-random manner, which in turn reduces genetic diversity in the entire locus (Cadzow et al., 2014). Therefore, in light of the non-random association of the alleles associated with COVID-19 with their neighboring alleles, we can provide you with useful contextual information on seeing the pattern of positive selection in different human populations and the selective advantage it might be imparting on a certain population.

In the wake of a pandemic, two significant GWAS studies have been put forth that have successfully associated five genetic loci with COVID-19 severity. In this work, three out of five SNPs (also SNVs) associated with COVID-19 severity lie in or within the close proximity of immunity-related genes were focused on from an evolutionary perspective (see methods). The shortlisted three SNPs in this study are a result of a GWAS conducted on 2244 critical care patients with COVID-19 in the UK (Pairo-Castineira et al., 2020). The three novel COVID-19-associated SNPS are 1) rs10735079 in gene cluster of *OAS1, OAS2* and *OAS3*, 2) rs2109069 within *DPP9* near gene encoding tyrosine kinase 2 (*TYK2*) and 3) and rs2236757 in the interferon receptor gene *IFNAR2* (Pairo-Castineira et al., 2020).

**FIGURE 1:**
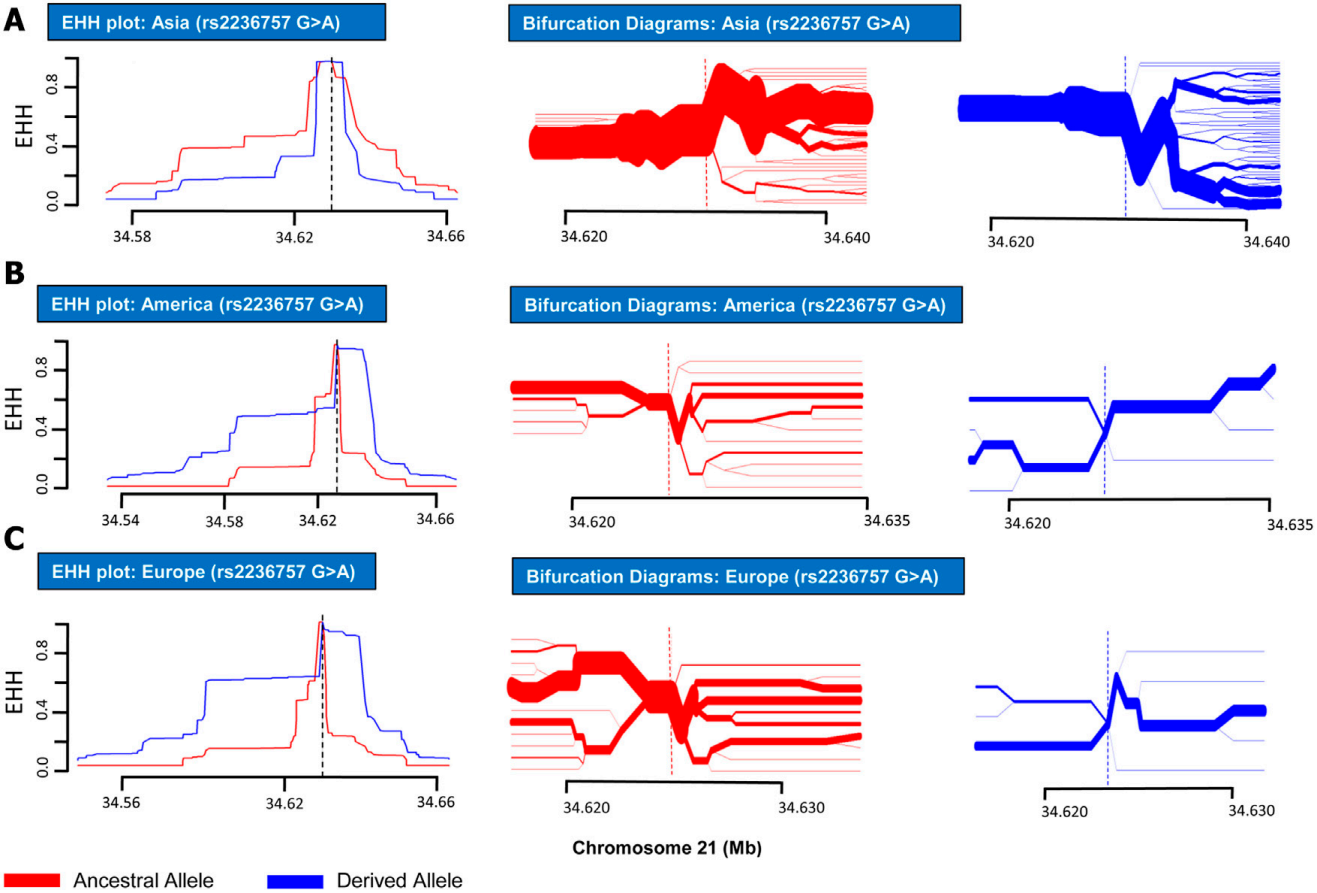
EHH plots and Bifurcation Diagrams for SNP rs2236757 (in non-African Populations). EHH plots and Bifurcation Diagrams for SNP rs2236757 in Asian **(A)**, American **(B)** and European **(C)** populations. EHH = l on Y-axis indicates all haplotypes carrying either ancestral or derived state of the allele are matching upto this point. X-axis contains coordinates for human chromosome 21. Ancestral allele is shown before the derived allele, separated by a “>” symbol. In the EHH plots, smaller area under the curve for both ancestral and derived alleles (G > A) shows no signs of recent positive selection in any of the aforementioned populations.

**FIGURE 2:**
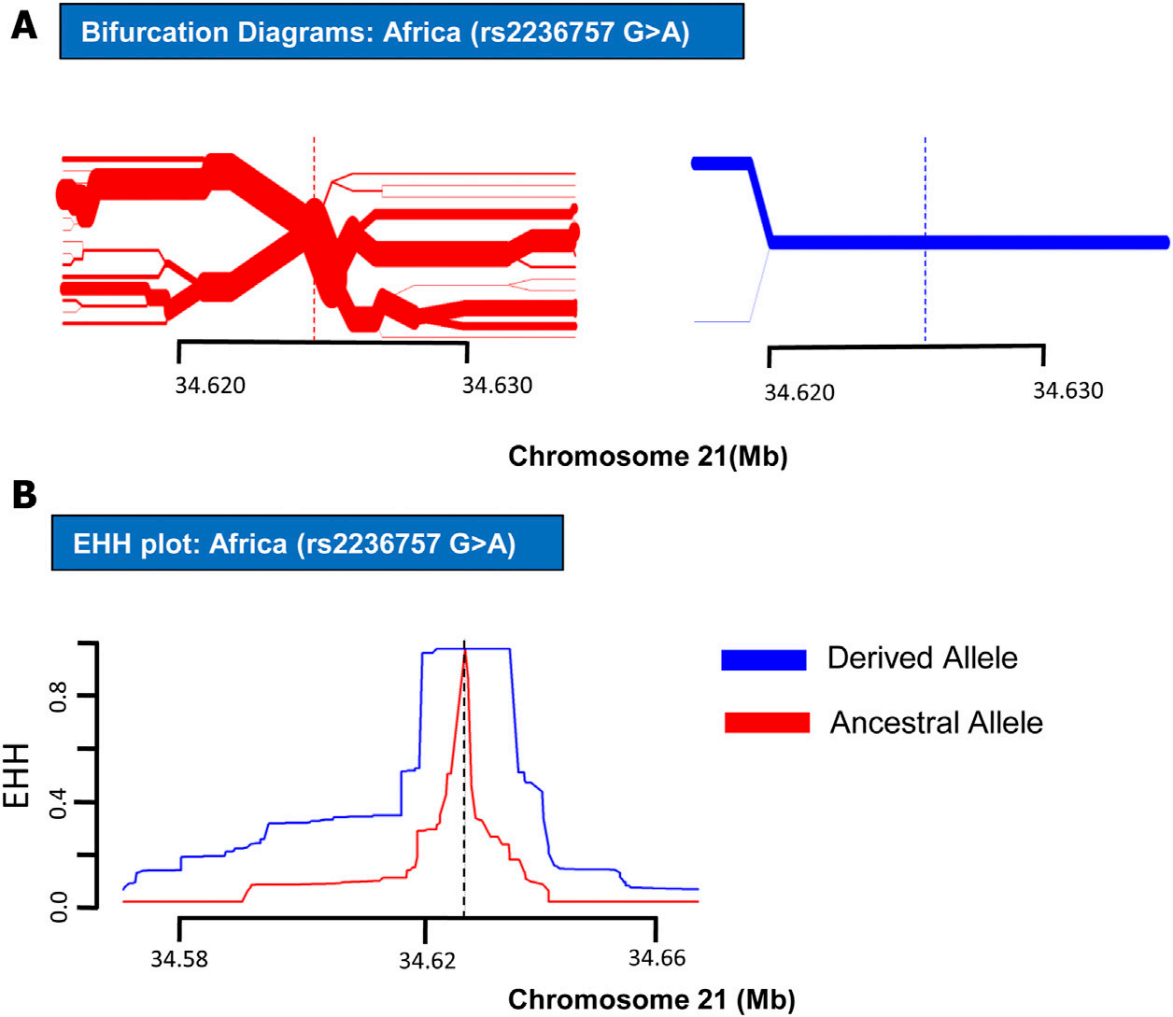
EHH plot and Bifurcation Diagrams for SNP rs2236757 (in African Populations). EHH plots and Bifurcation Diagrams of SNP rs2236757in African populations. **(A)** Bifurcation Diagram of the derived variant of the SNP rs2236757 (shown in blue) shows long haplotype and absolutely no branching at the nodes upto 14.6 kb region. **(B)** EHH plot for SNP rs2236757 shows derived allele A (shown in blue) is under positive selection. EHH = 1 indicates all haplotypes carrying either ancestral or derived state of the allele are matching upto this point.

In order to analyze positive selection on the aforementioned three SNPs, statistical approaches such as EHH tests and haplotype bifurcation diagrams were applied to the SNP data collected from the 1000 Genomes Phase III (Sabeti et al., 2002). By applying EHH tests and haplotype bifurcation diagrams, it was found that the derived minor allele “A” of SNP rs2236757 residing in the *IFNAR2* gene has undergone recent positive selection in the African population alone out of the four population categories (African, European, Asian, and American), whereas no positive selection signals were identified in any of the population categories for the ancestral major allele “G” of SNP rs2236757 (Figure 1). In 1322 haplotypes of samples of African individuals from 1000 Genomes Phase III, unbroken haplotypes, indicative of stronger linkage disequilibrium, were observed to be up to 15 kb in length at an EHH value of 1 for derived minor allele “A” of SNP rs2236757 (Figure 2). In LD analysis carried out at Ensembl, it was observed that the SNP rs2236757 is co-inherited with the neighboring SNP rs2073361 in CLM and MXL (America), with LD (*r* ^2^) of 0.8486 and 0.9394, respectively (Cunningham et al., 2015). The higher LD (*r* ^2^) values indicate that the two SNPs are in strong LD and one of them is the causal SNP for such a behavior. Moreover, in Haploreg, LD (*r* ^2^) was also observed to be 1 for the SNP rs2236757 inclusive of the neighboring SNPs up to the said ∼15 kb region in the African population, hence, indicating non-random association between the neighboring alleles and the SNP rs2236757 (Sabeti et al., 2002; Ward and Kellis, 2012). Furthermore, the F_st_ value of rs2236757 in the African population was calculated via VCFtools and observed to be 0.12. The F_st_ value higher than 0.1 is generally indicative of a significant high level of genetic differentiation between one population and the rest of the populations (Table 1) (Danecek et al., 2011). It is also interesting to note that the major allele “G” of SNP rs2236757 was found to be conserved in all of the 37 Eutherian mammalian species at Ensembl (Cunningham et al., 2015). On similar lines, EHH plots and haplotype bifurcation diagrams when applied to the remaining SNPs/SNVs rs10735079 and rs2109069 did not indicate longer, unbroken haplotypes of considerable length. Therefore, no positive selection signals were observed in any of the sub-populations on the respective derived and ancestral alleles of the SNPs rs10735079 and rs2109069 (Supplementary Figures S1, S2). A schematic flow of the results obtained can be viewed in Supplementary Figure S3.

**TABLE 1:** Weir and Cockerham F_st_ values evaluated for SNPs rs10735079, rs2109069 and rs2236757.

S.No.	SNPs	Genomic coordinates (hg19)	Genes	A > D^a^	Weir and cockerham F_st_			
					Africa	Asia	America	Europe
1	rs10735079	chr12: 113380008	OAS1, OAS2, OAS3	A > G	0.025	0	0	0.039
2	rs2109069	chr19: 4719443	DPP9	A > G	0.0007	0.008	0	0.054
3	rs2236757	chr21: 34624917	IFNAR2	G > A	**0.120**	0.075	0.007	0.020

a The bold value indicate a higher Weir and Cockerham F_st_ value for SNP rs2236757 in the African population

The evolutionarily selected interferon (IFN)-mediated innate immune response is inbred in genomes and provides a powerful initial line of defense against invading pathogens (Schneider et al., 2014). Type 1 IFNs comprise the largest class that exhibit varied binding affinity with the IFNAR1/2 receptor complex and as a result diversified anti-viral responses are induced and amplified in the host (Moraga et al., 2009). In a recent cohort-based study, pulmonary tissue samples from the severely affected patients of COVID-19 and pH1N1 influenza showed differential expression of two genes, *IFI27* and *IFI6,* both belonging to type 1 IFNs (Kulasinghe et al., 2021). The findings for differential expression of the *IFN* genes controlling the immunoregulatory responses have also been corroborated in transcriptomic profiling of the hospitalized COVID-19 patients (Ahern et al., 2021). In most cases of COVID-19 patients, genetic aberrations in antiviral innate immune interferon (IFN) loci and dysregulation of IFNs have also been correlated with the severity of the SARS-CoV-2 infection (Lopez et al., 2020).

IFNAR2 is a subunit of the type 1 IFN receptor complex. Upon binding of type 1 IFNs with the surface receptor complex, JAK kinases are induced along with the activation of STAT transcription factors, which in turn initiate the transcription of the immune response genes (Saleh et al., 2004). In recent GWAS studies, polymorphisms in the *IFNAR2* gene have shown a direct association with COVID-19 hospitalizations (Smieszek et al., 2021). IFNAR2 protein has also been nominated along with ACE2 as drug targets for expedited clinical trials (Gaziano et al., 2021). In summary, our results have indicated recent positive selection on derived risk allele “A” of SNP rs2236757 within the *IFNAR2* gene in the African population in the shape of a long, unbroken ∼15 kb haplotype (Figure 2). However, it has been established that some risk alleles may be positively selected individually or as part of an underlying biological function because of a currently unknown advantage they may have imparted on the host genome (Corona et al., 2010). In spite of the presented data, because of the dubious nature of COVID-19 spread among different populations in the face of the emerging new variants, it is not yet conclusively possible to point out a population which could be at a selective advantage and therefore with a lower mortality rate due to COVID-19. Nonetheless, the identified positive selection on a risk allele of SNP rs2236757 in the intronic region of the *IFNAR2* gene holds importance. This study confers the idea that natural selection within immunity-related can be used as a tool in addressing the symptomatic idiosyncrasy of the current COVID-19 pandemic. Moreover, the results also highlight the need for more GWAS studies inclusive of diverse population data and subsequently extensive assessment of the genetic aberrations that can be done under the light of evolution to understand the heterogeneous severity pattern of COVID-19 among different human populations.

## Data Availability

The original contributions presented in the study are included in the article/Supplementary Material, further inquiries can be directed to the corresponding author.

## Abbreviations

EHH, extended haplotype homozygosity; GWAS, genome wide association study; LD, linkage disequilibrium; SNV, single nucleotide variant.
