# Supplementary material for: An Evolutionary Insight Into the Heterogeneous Severity Pattern of the SARS-CoV-2 Infection
Source: Front Genet. 2022 Mar 22;13:859508. doi: 10.3389/fgene.2022.859508 (PMC8981084; doi:10.3389/fgene.2022.859508)

Fig. S1. EHH Plots and Bifurcation Diagrams of SNP rs10735079

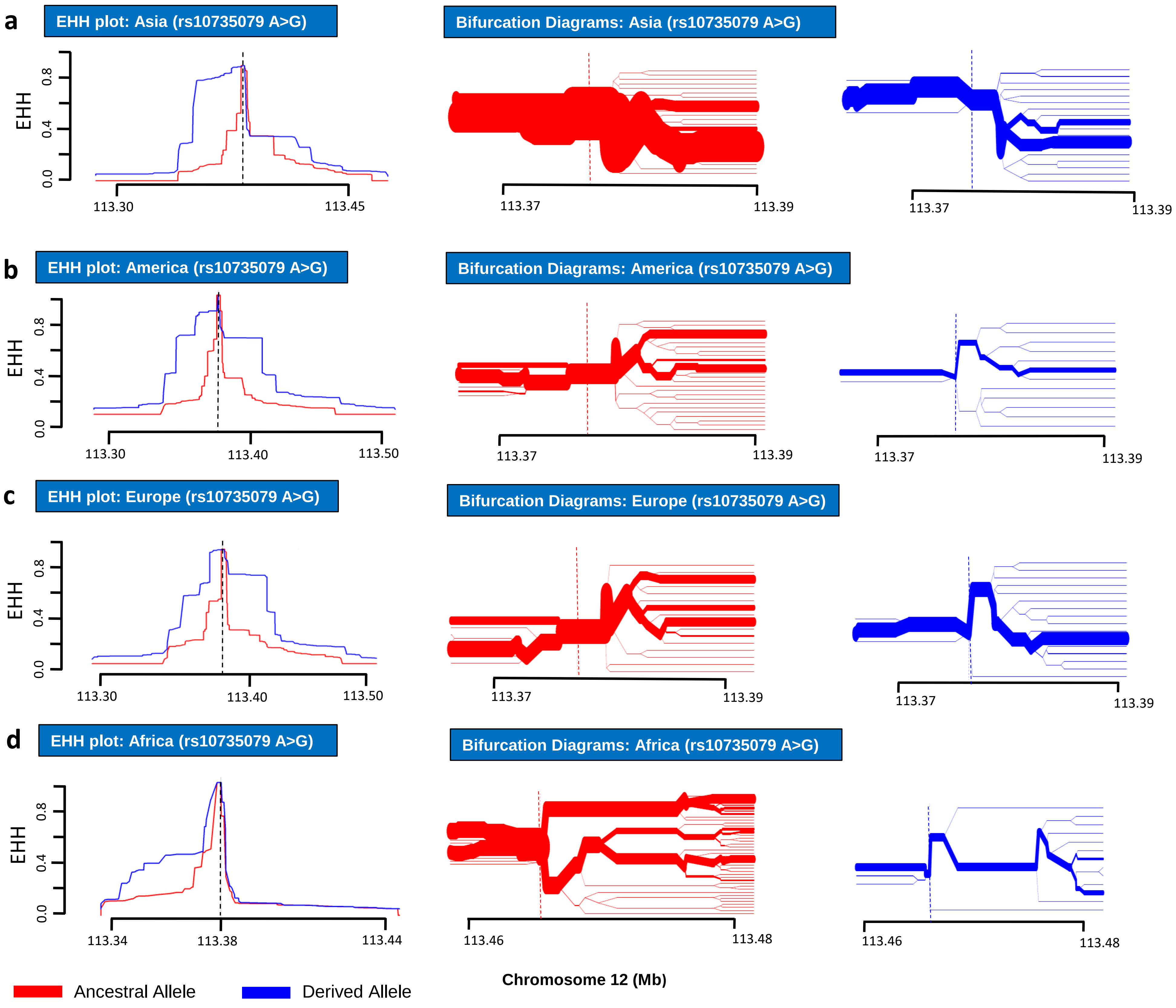

Fig. S1. (a-d) depicts EHH plots and Bifurcation Diagrams for SNP rs10735079 in Asian, American, European and African populations. EHH=l on Y-axis indicates all haplotypes carrying either ancestral or derived state of the allele are matching upto this point. X-axis contains coordinates for human chromosome 12. Ancestral allele is shown before the derived allele, separated by a ">" symbol. In the EHH plots, smaller area under the curve for both ancestral and derived alleles (A>G) shows no signs of recent positive selection in any of the populations.

Fig. S2. EHH Plots and Bifurcation Diagrams of SNP rs2109069

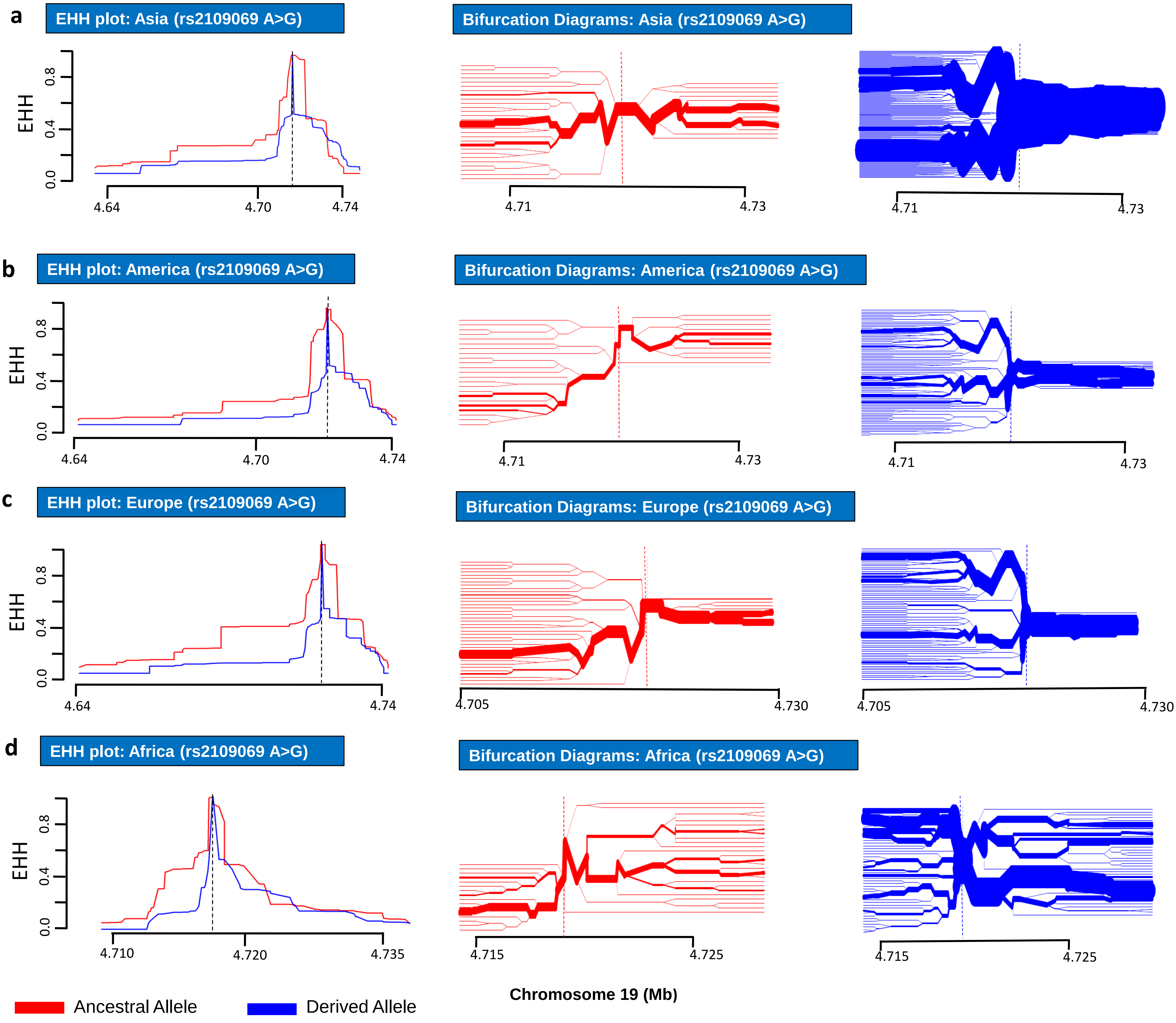

Fig. S2. (a-d) depicts EHH plots and Bifurcation Diagrams for SNP rs2109069 in Asian, American, European and African populations. EHH=l on Y-axis indicates all haplotypes carrying either ancestral or derived state of the allele are matching upto this point. X-axis contains coordinates for human chromosome 19. Ancestral allele is shown before the derived allele, separated by a ">" symbol. In the EHH plots, smaller area under the curve for both ancestral and derived alleles (A>G) shows no signs of recent positive selection in any of the populations.

Fig. S3. A schematic flow of implemented steps in the methodology.

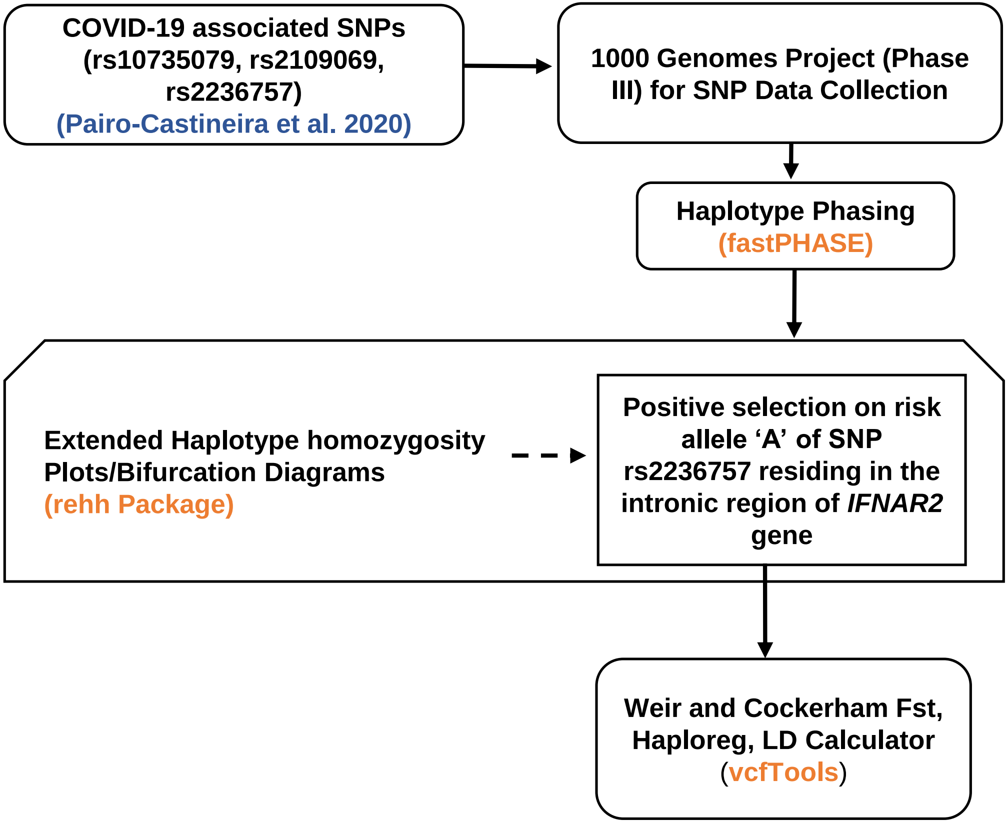

Supplement: Supplementary file 1 [file DataSheet1.PDF]
